# Role of rs193922155 in the etiopathogenesis of osteogenesis imperfecta with description of the phenotype: A case report

**DOI:** 10.1097/MD.0000000000027021

**Published:** 2021-08-27

**Authors:** Janusz Płomiński, Marek Szwabowicz, Ewa Fiedorowicz, Roman Grzybowski, Maria Latacz, Anna Cieślińska

**Affiliations:** a Clinical Department of Trauma-Orthopedic Surgery and Spine Surgery of the Provincial Specialist Hospital, Olsztyn, Poland; b Department and Clinic of Orthopaedics and Traumatology, Collegium Medicum, University of Warmia and Mazury, Olsztyn, Poland; c Faculty of Biology and Biotechnology, University of Warmia and Mazury, Olsztyn, Poland; d Faculty of Medicine, Collegium Medicum, University of Warmia and Mazury, Olsztyn, Poland.

**Keywords:** case report, collagen type I alpha 1gene, osteogenesis imperfecta, single nucleotide polymorphism

## Abstract

**Introduction:**

Osteogenesis imperfecta (OI) is a disorder of the connective tissue that mainly causes the bones to become excessively brittle. The vast majority of OI cases are associated with mutations in the genes encoding the I alpha.

**Patient concerns:**

A 57-year-old woman office worker was admitted because of severe, long-lasting pain in the thoracic spine while bending down. She and her daughter have a history of multiple atraumatic fractures form early childhood.

**Diagnosis:**

Both women were pre-diagnosed with OI based on their phenotype. The genetic testing has shown single nucleotide polymorphism (rs193922155) in the gene encoding the collagen type I alpha 1 which until now was only likely pathogenic.

**Interventions:**

Bone mineral density measurement revealed osteoporosis. The mother was prescribed with Vitamin D3 and calcium supplementation, but the daughter does not take any medication. The mother had vertebroplasty performed because of Th 9–12 vertebral body compression fractures. The cardiovascular diseases, spontaneous hematomas, joint dislocations were excluded.

**Outcomes:**

For mother postoperative pain reduction was achieved.

**Conclusion:**

To the best of our knowledge, this is the first publication that confirms the pathogenic effect of this mutation and describes the phenotype.

## 1 Introduction

Osteogenesis Imperfecta (OI) is clinically and genetically heterogeneous group of heritable connective tissue disorders characterized by an inherited skeletal dysplasia with susceptibility to bone fractures, blue sclera, bone deformity, and relaxation of skin, with variable degree of severity and presumed or proven defects in collagen type I biosynthesis.^[1,2]^ The incidence of OI is 1/15,000 to 20,000 birth.^[3]^ The main problem of this disease is excessive bone fragility which varies depending on the type of OI.^[4]^

The current classification (Nosology and classification of genetic skeletal disorders: 2019 revisiom) differentiate OI into five groups with different course of the illness.^[5,6]^ Within different types of OI, type I is the most common, accounting for approximately 70% of OI incidence (Swedish population, only type I, III, IV were taken into consideration). ^[7]^

Great majority of OI are generated by monoallelic mutations in collagen type I alpha 1(*COL1A1)* or collagen type I alpha 2 (*COL1A2)* – de novo or inherited in an autosomal dominant manner.^[8]^

In this publication, we present a case of two related women with mutation c.370-2A>G in *COL1A1* gene. Mutation c.370-2A>G is a single nucleotide polymorphism (SNP) and the nomenclature appropriate for SNP is rs193922155. Until now it has been considered as only potentially pathogenic.^[9]^ To the best of our knowledge, this is the first publication that confirms the pathogenic effect of this mutation.

## 2 Case presentation

The participants of this study were mother and daughter from a Polish family (Caucasian race) with OI who were treated at the Clinical Department of Trauma-Orthopedic Surgery and Spine Surgery of the Provincial Specialist Hospital in Olsztyn. Both participants gave informed consent to the study, which was performed with maintained ethical standards as well as certified by the Local Bioethics Commission at University of Warmia and Mazury in Olsztyn (49/2019). The blood samples were collected for genetic testing in NZOZ Genomed (Poland) using Next Generation Sequencing for collagen genes analysis.

**Figure 1:**
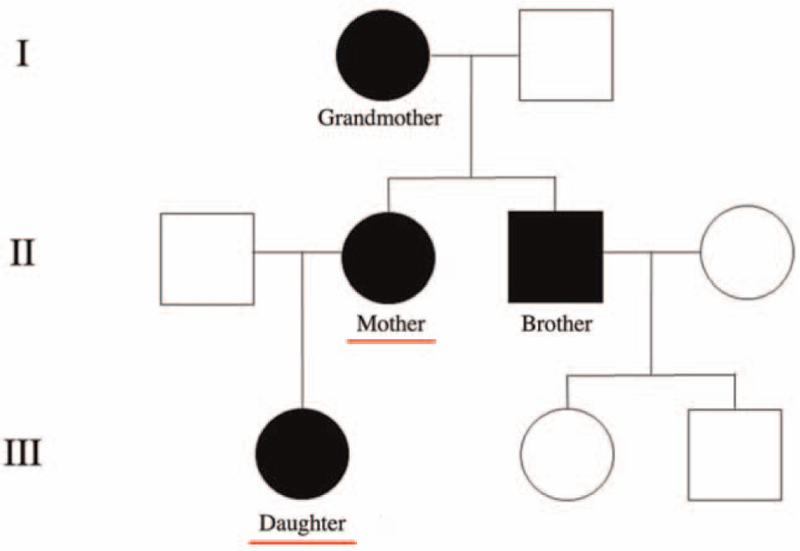
A family pedigree. The full black symbol indicates members of family with osteogenesis imperfecta; white symbols represent unaffected individuals.

Their medical histories information was collected to generate a family pedigree (Fig. 1.) and describe the phenotypes.

### 2.1 The mother – detailed information

The mother is 57-year-old office worker. She was diagnosed as a child with OI (without any genetic testing until now). For several months, she suffered from severe pain in the thoracic spine while bending down. From the age of five, several fractures of the shafts of long bones occurred and they were not treated surgically. Subsequent fractures were not associated with any injuries and simultaneously the number decreased with age. Neither cardiovascular disease was diagnosed, nor spontaneous hematomas and joint dislocations were reported. Vitamin D3 and calcium supplementation was prescribed. Pain in the musculoskeletal system: 5 (according to the Visual Analogue Scale). Vertebroplasty was performed because of Th 9–12 vertebral body compression fractures. Postoperative pain reduction was achieved.

### Planned treatment

Nearly all the mother's fractures to date have been treated non-operatively. Due to the fracture of the spine with its deformation, surgical treatment was planned. After CT and MRI examination, the patient was qualified for vertebroplasty. The postoperative course was uneventful. The patient is wearing Jewett's corset. Due to confirmed osteoporosis, the patient was prescribed with pharmacological treatment. Still under orthopedic control on an outpatient basis.

### 2.2 The daughter – detailed information

The daughter is 32-year-old waitress with prediagnosed OI (again without any genetic testing until now). Like mother, at the age of five the first fracture happened and in subsequent years more fractures appeared with no obvious traumatic cause. Osteoporosis was confirmed with bone mineral density measurement. There were no deformities of the spine and limbs. Pain in the musculoskeletal system: 6 (according to the Visual Analogue Scale). She does not take any medications. In 2012 a surgical intervention was performed - traumatic multifragmentation fracture of the kneecap.

### Planned treatment

Nearly all the daughter's fractures were treated non-operatively. Satisfactory treatment results without lower limb deformities. There was no axis disturbance or limitation of mobility in joints. Due to confirmed osteoporosis, she was qualified for pharmacological treatment. Moreover, due to periodic pain in the osteoarticular system, rehabilitation was recommended.

Characteristic of phenotype of the mother and her daughter were presented in Table 1.

**Table 1:** Characteristic of phenotype.

Family member	Mother	Daughter
Blue sclera	yes	yes
Short stature [cm]	153	168
Bone reduction	yes	yes
Dentine development disorders	no	no
Laxity of joints and ligaments	yes	yes
Tendency to bruise, skin thinning	yes	yes
Hearing loss	yes	yes
Genetic test	a heterozygous c. 370–2A>G	a heterozygous c. 370-2A>G

The recommended treatment for OI fractures is non-operative treatment. When immobilization is used, attention is paid to local complications in the form of pressure ulcers, circulatory disorders. Surgical treatment is indicated only in cases of large deformities in the form of shortening or rotation disorders that may lead to non-union or limb dysfunction. However, in the case of fractures in children, K-wire stabilization or minimally invasive procedures are recommended.

Nearly all fractures in both patients were treated non-operatively and healed without excessive callus. Fracture healing time did not exceed 3 months. The maternal Th9-Th12 vertebral fracture was treated surgically, vertebroplasty. Pain was reduced and the spine functioned well. No further fractures were observed 6 months after the procedure. Radiological examinations showed no deterioration in the sagittal balance of the spine. The care plan involves careful monitoring of both patients. The cardiovascular diseases, coagulation disorders, problems with joints have already been excluded. They are both being encouraged to take up moderate physical activity. It is extremely important to educate the daughter since her prospective offspring has 50% of chance to get OI.

Both the brother (53 years) and the grandmother (81 years) were diagnosed with OI (Fig. 1), but without any genetic testing. Over the years, multiple fractures were noted and currently they have osteoporosis.

## 3 Discussion

Detected mutation in two individuals was reported in ClinVar database as potentially pathogenic in August 2011.^[9]^ There is no publication in the PubMed database referring this SNP.^[10]^ G allele is potentially responsible for the disruption of splicing as it is a variant within 3’ end of the intron.^[10]^ Genetic alteration in splice acceptor or donor can cause exon skipping, short deletion as well as insertion in mature mRNA.^[11]^

The diagnosis was provided on the basis of clinical symptoms and repeated incidents of spontaneous fractures. The clinical observation shows that the patients in the study qualify for type I OI. Lack of limb deformation healing of fractures without hyperplastic callus and progressive scoliosis confirm these observations. The performed genetic tests were to attribute the detected mutation to the clinical course of the disease. In addition, we consider the usefulness of examining family predisposition and the presence of gene mutations in offspring and the assessment of OI occurrence to be clinically significant. Early detection of a genetic defect, especially in children, might reduce the risk of fractures and deformities by undertaking targeted treatment and rehabilitation.^[12]^

In the case our patients, there is no short stature and low BMI, which is emphasized in all types of OI, because OI is usually associated with low bone density. Radiological symptoms of osteoporosis, especially in the case of a group of young patients, must lead to the suspicion of hormonal disorders and OI. Genetic testing can be useful when OI clinical manifestation is not obvious. It is claimed that the clinical management of OI depends on the severity of the phenotype, and in uncomplicated OI type I physical activity may be similar to the general population.^[13]^ Moreover, treatment may be limited to fracture management, the primary goal of which is medical follow-up to detect complications such as vertebral compression fractures.^[14]^

85% patients with OI have a disease-causing mutation in one of the two genes encoding the type I alpha chains of collagen, COL1A1 and COL1A2.^[15]^ Type I collagen is a major component of the organic matrix of the bone, in OI the fibrils are thinner than the normal ones so the bone is brittle.^[16]^ According to Tauer el al., almost all individuals with a typical clinical manifestation of type I have a detectable mutation in one of the OI related genes currently known.^[17]^ A recent sequencing study in nearly 600 people with a clinical diagnosis of OI found a disease-causing mutation in 97% of patients with type I OI (all of whom had *COL1A1* or *COL1A2* mutations) and 99% of those with more severe types of OI.^[18]^ More than 40% of the mutations, that were found in *COL1A1* or *COL1A2*, were novel mutation.^[19]^

## 4 Conclusions

Low-energy fractures in children or successive fractures at short intervals require diagnosis for OI. The variety of clinical types of OI in correlation with genetic testing enables an early diagnosis and determines the prognosis. We have confirmed the pathogenic role of rs193922155 in OI, highly probable type I which is inherited in an autosomal dominant manner. Explaining the disease-causing mutation is useful in patients with a clinical diagnosis of OI because it provides information about the risk of relapse in the family and allows the identification of affected family members.

## Acknowledgments

The authors would thank the members of Family, who participated in the research.

## Author contributions

**Conceptualization:** Janusz Płomiński, Marek Szwabowicz, Roman Grzybowski.

**Data curation:** Ewa Fiedorowicz.

**Methodology:** Anna Cieślińska.

**Project administration:** Janusz Płomiński, Anna Cieślińska.

**Supervision:** Janusz Płomiński, Anna Cieślińska.

**Visualization:** Ewa Fiedorowicz, Maria Latacz, Anna Cieślińska.

**Writing – original draft:** Janusz Płomiński, Ewa Fiedorowicz, Maria Latacz, Anna Cieślińska.

**Writing – review & editing:** Janusz Płomiński, Ewa Fiedorowicz, Maria Latacz, Anna Cieślińska, Marek Szwabowicz, Roman Grzybowski.
